# Experimental and Numerical Calculation Study on the Slope Stability of the Yellow River Floodplain from Wantan Town to Liuyuankou

**DOI:** 10.3390/toxics11010079

**Published:** 2023-01-14

**Authors:** Hao Wang, Qing Hu, Weiwei Liu, Liqun Ma, Zhiying Lv, Hongyu Qin, Jianbo Guo

**Affiliations:** 1 School of Civil and Architectural Engineering, Henan University, Kaifeng 475004, China; 2 Engineering Technology Research Center for Embankment Safety and Disease Control, Ministry of Water Resources, Zhengzhou 450003, China; 3 The College of Geography and Environmental Science, Henan University, Kaifeng 475004, China; 4 College of Science and Engineering, Flinders University, Adelaide 5042, Australia

**Keywords:** floodplain, slope, scour, flume experiment, numerical calculation

## Abstract

More than two million people live on the floodplains along the middle and lower streams of the Yellow River. The rapid development of industry and agriculture on both sides of the Yellow River has caused serious pollution of the floodplain soil. Erosion by water has led to the destruction of the floodplain which has not only compressed people’s living space but also resulted in a large amount of sediment containing heavy metals entering the river, aggravating water pollution. To further study the law governing the release of pollutants in soil, this work, based on field surveys of the Yellow River floodplain slopes from Wantan town to Liuyuankou, was focused on determining the failure mechanism and laws for the floodplain slope through the combination of a flume experiment and numerical calculations. The results showed that the floodplain slopes, composed of clay and silty sand, presented an interactive structure. Under the action of water erosion, the slope was first scoured to form a curved, suspended layer structure, and then the upper suspended layer toppled. The bank stability coefficient decreased by about 65% when the scour width increased from 0.07 m to 0.42 m, and the water content increased from 20% to 40%. For the failure characteristics, the angle of the failure surface was negatively correlated with the scour width, and the distance from the top failure surface to the bank edge was about 2.5 times that of the scour width.

## 1. Introduction

Pollution caused by heavy metals in soil is attracting more and more attention. The rapid development of industry and agriculture along the lower Yellow River has increased the number of heavy metals in the soil [1,2]. When the heavy metal content in bank soil accumulates to a certain extent, it causes damage to riparian organisms, and the numbers of plant species significantly decrease with increases in the heavy metal concentration [3,4,5]. In recent years, the collapse of the bank has resulted in polluted soil continuously entering the Yellow River, causing serious pollution in the water body. In addition, some studies have found that the riverbank can intercept the polluted sediments carried by the upstream river, but when the riverbank is damaged, these pollutants re-enter the river [6,7]. Zhao surveyed ten major tributaries along the middle and lower reaches of the Yellow River and found that all of the lower tributaries had poor water quality and that the pollutants mainly came from industry and agriculture [8]. Walling found that 90 percent of heavy metals in the water were associated with suspended solids and sediments [9]. Many scholars have studied the pollution of the Yellow River [10,11]. However, there is little research on the release law for soil pollutants resulting from erosion. This work was undertaken to lay a foundation for further research on the release of pollutants by studying the failure law and failure rate for the slope of the Yellow River floodplain.

The Yellow River is about 5464 km long and is one of the longest rivers in the world [12]. Xiaolangdi Dam is the last cascade reservoir in the main stream of the Yellow River and is located at the exit from the last gorge in the middle stream of the Yellow River. The important roles of the dam are to store water and hold back sand. Compared to the average from 1956 to 2016, the measured annual sediment transport volume at Xiaolangdi decreased by 90.5% in 2021 [13]. As the sediment entering the downstream channel has been greatly reduced, the sand-carrying capacity of the current cannot be satisfied, and the downstream riverbank is being scoured [14,15,16,17]. The continuous collapse of the slope is causing a number of environmental problems and increasing the risk of damage to infrastructure along the riverbank [18,19,20]. It is expected that the lower reaches of the Yellow River will continue to be strongly scoured, with the concave bank being the most strongly effected, followed by the straight bank.

Research on failure mechanisms has developed from homogeneous to multi-layer slopes. Das and others studied the scour damage in cohesive homogeneous slopes [21,22,23]. Yu conducted scouring experiments on non-cohesive and cohesive homogeneous banks, revealing the iterative cycle of bank erosion and riverbed deformation [24]. Hazari et al. analyzed the stability of two different cohesive soil slopes [25,26,27]. Dapporto studied the failure mechanism in a riverbank with a dual structure of sand and clay [28]. Yodsomjai studied the stability of double-layer conical slopes [29]. Li evaluated the stability of a three-layer slope composed of two cohesive soil layers and backfill soil [30]. However, previous studies on stratified slopes rarely go beyond three layers, while the floodplain slopes of the lower Yellow River show an interactive structure because the numerous floods have resulted in the silty sand and clay carried by the river being alternately deposited on the river beach. Although the Yellow River floodplain is rapidly collapsing, the failure pattern and characteristics of the floodplain slope composed of silty sand and clay interlayers have not been extensively studied. The slope of the Yellow River floodplain presents a typical interactive stratified structure, and slopes with special structures have not been widely studied. Gusman suggested that one of the important factors in slope stability analysis is water content [31]. Hooke and Casagli et al. stated that soil moisture content is essential to beach erosion [32,33,34]. Gu highlighted the influence of different irrigation methods on landslides [35]. In light of this work, it is necessary to consider the influence of water content in the research process.

There has been much research undertaken on river scour [36,37]. Pandey studied the erosion of piers by water flow [38]. Huang established a model for sediment transport caused by flooding [39]. Yang studied the operational impact of the Three Gorges Dam on river scour [40]. Yan et al. studied the protective effect of vegetation on gully banks through scouring experiments [41]. Another study discussed the sediment transport trajectory [42]. Dey conducted a scouring experiment with a bend flume and found that cross flow is an important factor in bank erosion [43]. Limit analysis theory with strict upper- and lower-limit solutions is often used in slope stability research [44,45]. Rao proposed a three-dimensional slope stability analysis method based on limit analysis [46]. Maghous analyzed the stability of rock slopes [47]. Huang used the upper limit theorem from limit analysis to draw a hazard map of shallow landslides [48]. Vasquez used a two-dimensional model to simulate scour and deposition along curved channels [49]. Karssenberg studied sediment transport in flood plains using a three-dimensional numerical model [50]. It can be seen from the previous studies that the research methods used are mainly numerical simulation and experimental methods. In this paper, soil collected from a field investigation was used to conduct a reduced model erosion test to study the failure mechanism for the floodplain slope. However, the physical model test is easily limited by size, resulting in different results [51,52]. Although parameters can be set according to different situations in the numerical simulation, the actual working conditions are complex [53,54]. In this paper, to take full advantage of both methods, the flume experiment and numerical calculation are combined to study the failure mechanism and pattern of floodplain slope. To make the conditions of the experiment closer to those of the slope in its natural state, soil collected from field investigation is used.

## 2. Materials and Methods

### 2.1. Study Area

**Figure 1:**
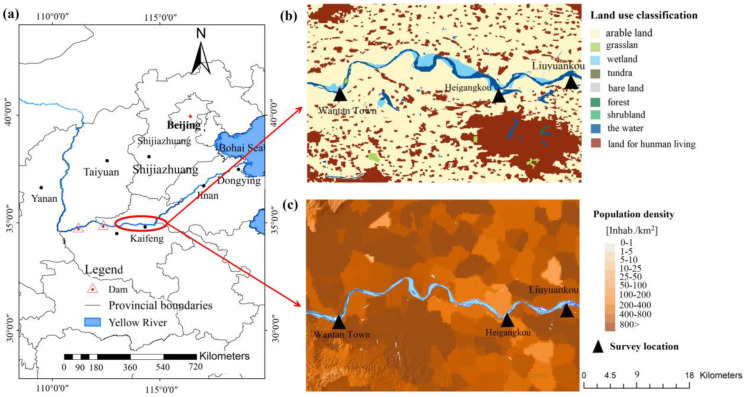
The location of the study area: (**a**) specific locations of the study area; (**b**) land use classification of the study area; (**c**) population density of study area.

The study area is located between Zhengzhou and Kaifeng (113°58′~114°23′ E, 34°50′~35° N), the core development cities of the Central Plains, and is an important agricultural planting area (Figure 1) [55,56]. Due to the flat terrain, sediment carried by the Yellow River from the upper reaches is constantly deposited in the area, forming the famous overhanging river. The climate in this region is a temperate monsoon climate, characterized by cold and dry winters and high temperatures and rain in summer. The average annual precipitation and temperature are 636 mm and 15 °C, respectively, and this area is rich in biological resources, with nearly 800 and 60 kinds of plants and animals, respectively. The economic development of the region is dominated by traditional industries, such as the chemical and energy industries, and crop cultivation, which have a significant impact on the ecological environment of the lower Yellow River [57]. Cultivated land is widely distributed in this area, and the soil is mainly loam, clay, and silt. However, due to the influence of climate and precipitation, wheat and corn are the main crop in winter and summer, respectively. Meanwhile, the sediment from the Loess Plateau is deposited to form floodplains which also have become significant land resources for agriculture. However, due to the operation of the Xiaolangdi Dam, the floodplain keeps retreating. Wang analyzed satellite images of the Yellow River from Huayuankou to Liuyuankou and found that nearly 32.08 km^2^ of farmlands on the floodplain were destroyed over 13 years [58].

### 2.2. Field Investigation

**Figure A1:**
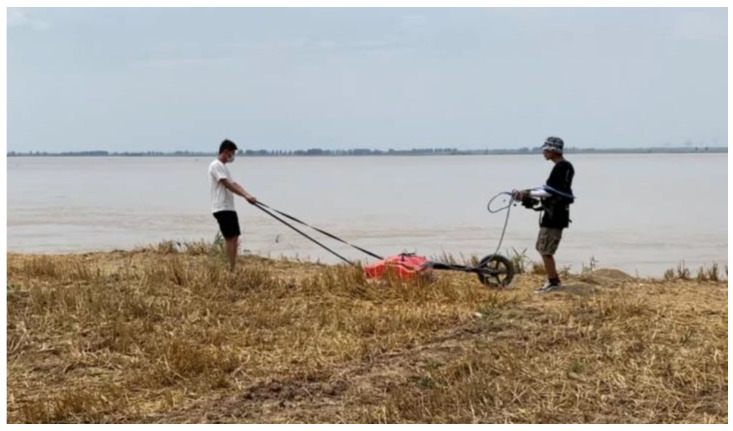
Scanning the floodplain in Wantan Town using the geological radar.

Thorne and Xia found that the shear strength reflects the slope stability [59,60]. To better understand the floodplain soil structure and mechanical properties, a field investigation, including soil sampling and scanning with SIR4000 geological radar, was carried out on the floodplain slopes from Wantan Town to Liuyuankou. Simultaneously, according to the erosion situation in the area, an investigation point was selected in Wantan Town, Heigangkou, and Liuyuankou, respectively (Figure 1). As shown in Figure A1, the geological radar was used to scan the floodplain slope parallel to the failure surface, and a total of 18 cross-sections from Wantan Town, Heigangkou, and Liuyuankou were swept. Given the actual situation of the floodplain, the sampling points were selected in the places where the collapse is prominent, and six cross-sections were selected for sampling (Table A1). Layered sampling was used, as the floodplain slope presents an interactive structure. To correspond to the scan results of geological radar, the sampling position and altitude were recorded strictly for each sampling. Undisturbed soil was sampled with a ring knife and sealed with plastic film, then placed in a plastic box. Additionally, the scattered soil was placed in a well-sealed plastic bag.

### 2.3. Laboratory Tests and Soil Types

The laboratory test is a standard method for classifying soil and understanding the physical and mechanical properties of soil, and the test procedure followed the geotechnical test method standards strictly [61]. The contents measured mainly included moisture content, density, specific gravity, shear strength index, and plasticity index.

### 2.4. The Scouring Experiment

**Figure 2:**
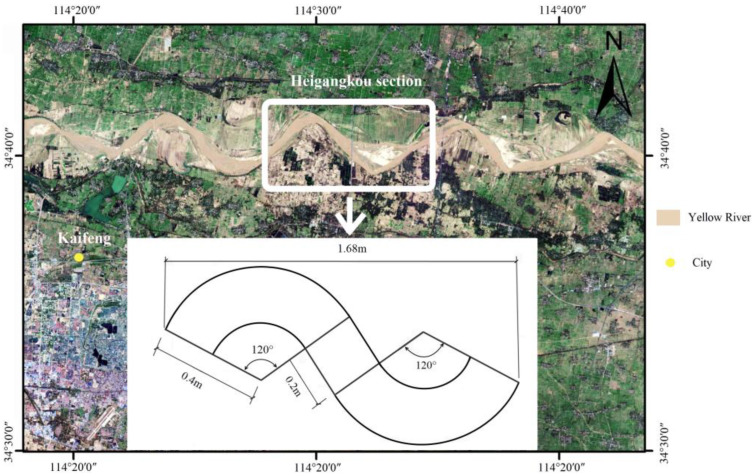
Schematic diagram of flume size.

The collapse of the floodplain slope directly threatens the safety of the Yellow River levee. Additionally, Heigangkou is located in Kaifeng, where levees have repeatedly broken in the past. The S-shaped flume following the actual bank size of the Heigangkou section was used to simulate the erosion process of the convex, concave, and straight banks, to study the failure pattern and mechanism of the floodplain slope (Figure 2).

**Figure 3:**
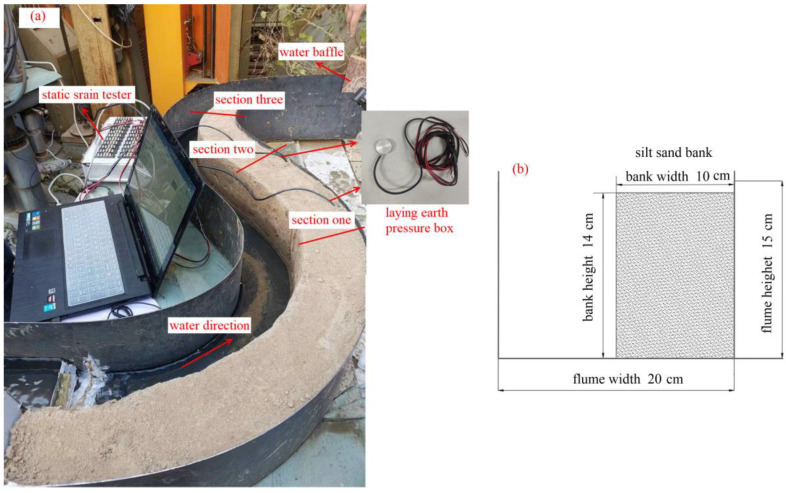
Actual diagram of the scouring experiment: (**a**) the layout of the scouring experiment; (**b**) side views of the flume.

Three profile positions were selected in this experiment, as shown in Figure 3a. A camera was set up on the convex, concave, and straight banks to capture the failure process. Earth pressure sensors were buried at the bottom of the concave and straight banks, and the measuring accuracy of the earth pressure box is 0–0.4 MPa. To prevent water from scouring the start and end of the slope, gravels were paved in the water inlet and outlet of the flume (Figure 3). The water outlet of the flume was provided with a water baffle which was used to control the water level height by changing its position at the same time (Figure 3a). The detailed description of the experiment equipment is shown in Table A2.

The river gradient and incoming water flow were taken as variables, and three working conditions were designed as a control. Each working condition was scoured for 1 h (the specific experimental design is listed in Table 1). Simultaneously, to simulate scouring damage when the water level changes, the water level was set to two heights (5 and 10 cm) and changed every 10 min. Each working condition required 75 kg of soil, collected from Wantan Town to Liuyuankou, and the size of the final experiment bank structure is illustrated in Figure 3b. The floodplain slopes present an interactive layered structure in their natural state; however, it was difficult to lay the clay directly. Therefore, the silty sand and clay were mixed as the experiment soil. To achieve the actual working condition density, it was necessary to tamp when laying the mixed soil.

### 2.5. Numerical Simulation

The limit analysis method was used to validate the failure pattern of a straight bank. Additionally, the effects of the scour width on the stability of the interactive layered slope were also analyzed. OptumG2 is a geotechnical software that integrates limit and finite element analysis and can simulate complex problems with nonlinear failure criteria [62,63,64,65]. Tschuchnigg and Sloan introduced the principle, advantages, and disadvantages of software in detail [45,66]. Every simulation in this paper required five iterations, and when the iteration number raised, the unit number increased from 5000 to 10,000.

## 3. Results

### 3.1. Field Investigation and Geotechnical Test Results

**Figure 4:**
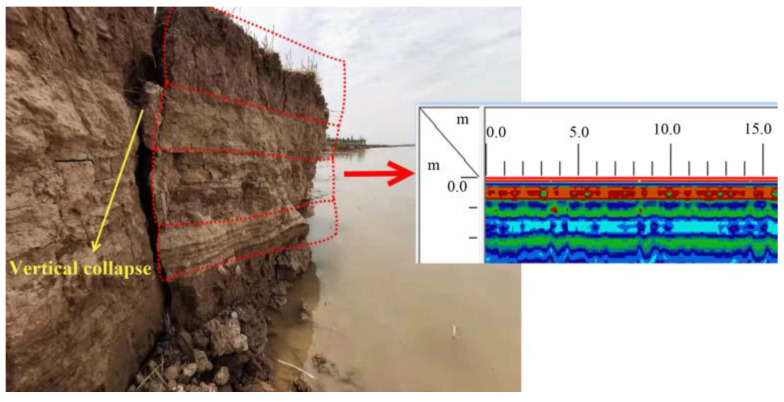
The scan result of a section in Wantan Town.

The bank presents a vertical stratified structure, and bank soil is roughly divided into brown and yellow soils (Figure 4). A scan result in Wantan Town is presented on the right side of Figure 4. Because different soils correspond to individual conductivity properties, nearly all the scan results of 18 sections showed a vertically layered structure.

**Figure 5:**
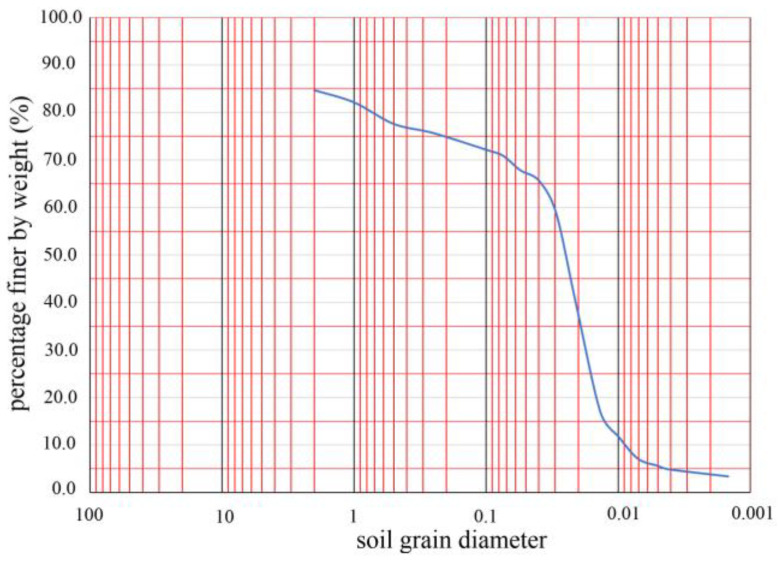
The particle gradation curve of the soil, whose scan color is blue.

Owing to the plasticity indexes above 17 (shown in Table 2), the soil was classified as clay in combination with relevant classification standards [67]. The yellow soil was first subjected to a sieving test, and then the particle gradation of the soil with particle size below 0.075 mm was determined by the densitometer method. Finally, the drawn particle gradation curve is shown (Figure 5). The particle size greater than 0.075 mm and smaller than 0.005 mm did not exceed 50% and 10% of the total weight, respectively. The plasticity index obtained from the liquid–plastic limit test was less than 10, so this soil was classified as silty sand according to the standard [67].

### 3.2. Scouring Experiment Result

#### 3.2.1. Failure Pattern

**Figure 6:**
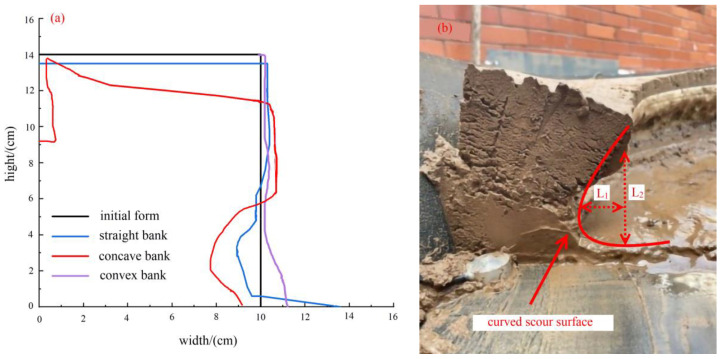
Bank failure pattern: (**a**) failure schematic diagram of slopes under working condition 2; (**b**) actual diagram of the concave bank under working condition 2 (L_1_: the scour height, L_2_: the scour width).

Figure 6a shows the bank failure schematic diagram of concave, straight, and convex banks under working condition 2. The erosion degree of the concave bank is greater than that of the straight bank and the convex bank, and the convex bank is silted. According to Figure 6b, it can be found that the slope toe was scoured to form an arc-shaped scouring surface, which was attributed to the presence of clay and the pseudo-cohesion of silty sand. To further understand erosion characteristics under the different variables and compare with previous studies, the scour height, L_1_ and width, L_2_, accurate to millimeters, were introduced.

#### 3.2.2. Variation of Scour Width and Height

**Figure 7:**
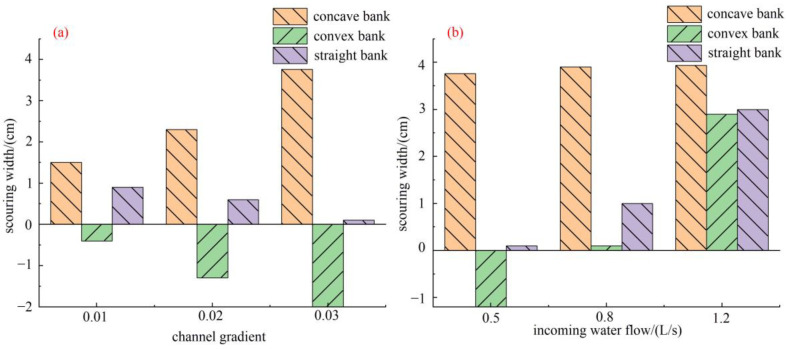
(**a**) The scour width varies when the channel ratio is a variable. (**b**) The scour width varies when incoming water flow is a variable.

Figure 7a shows that when the channel gradient equaled 0.01, the concave and straight banks were all eroded because of the weak circulation intensity caused by the low water velocity. As the channel gradient increased from 0.01 to 0.03, the scour width of the concave bank gradually enhanced. On the contrary, the convex bank which kept depositing from the channel gradient equaled 0.03.

Figure 7b shows that with the increase in incoming water flow, there was no deposition in the straight and convex banks. Wang found that when the water flow increased to a certain extent, the straight and convex banks were eroded [68].

#### 3.2.3. Variation of Earth Pressure

**Figure 8:**
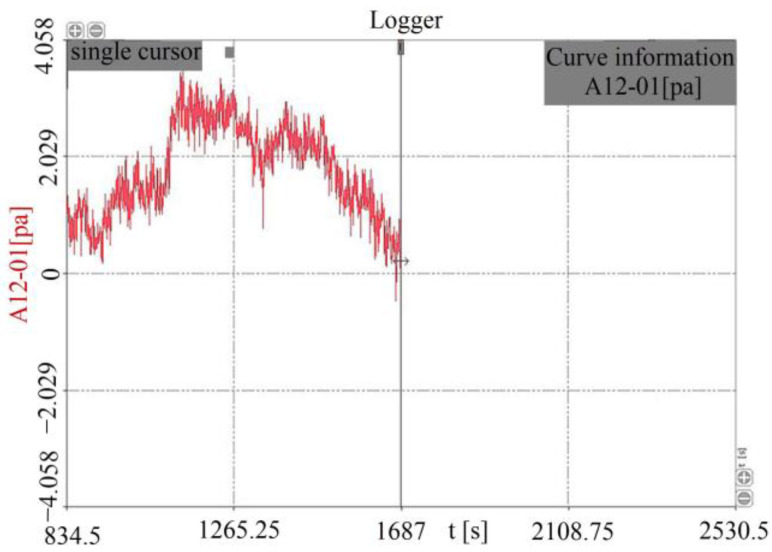
Earth pressure curve.

The change of the internal earth pressure from the experiment’s start to the experiment’s end is shown in Figure 8. Two factors affect earth pressure in the experiment process; earth pressure increases with an increase in soil water content, but water erosion decreases earth pressure. The earth pressure increased first and then decreased (Figure 8). This appearance indicated that the water content in the early stage had a more significant impact than water flow erosion. In the later period, the earth pressure decreased when the water content changed very little. In combination with the video recorded for the experiment, the slope collapsed when the earth pressure was less than before the experiment.

### 3.3. Numerical Results

#### 3.3.1. Establishing the Slope Model

**Figure 9:**
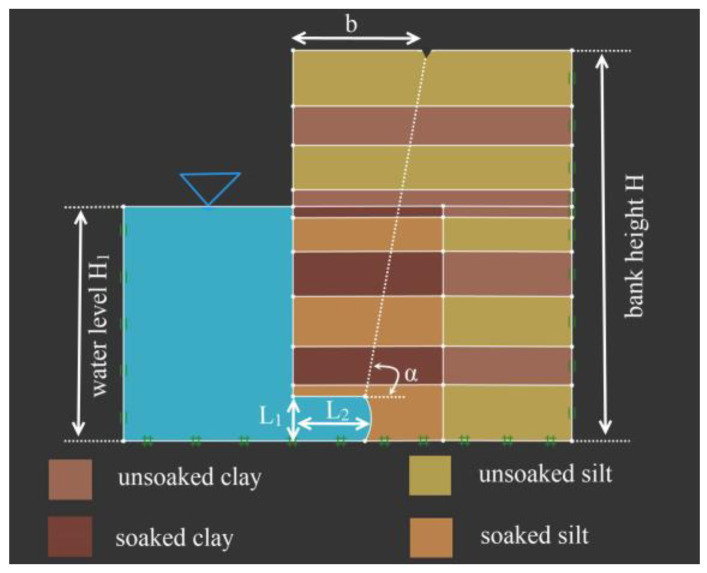
Generalized model of the bank collapse.

According to the flume experiment, the vertical slope formed an arc-shaped suspended layer under water flow scour. An equal proportional slope model, based on the size of the arc-shaped suspended layer, was established using OptumG2. A generalized model of slope collapse was established, wherein H, H_1_, b, and α represent bank height, water level, the length of the failure surface to the bank edge, and the failure surface angle, as seen in Figure 9.

There was a significant difference in the soil moisture content between the soaked part and the higher part of the floodplain slope during the flume experiment and field survey. To consider the effect of water content on the bank stability, the water content was divided into 20 and 40%, corresponding to two kinds of soil strength, according to the data measured in the experiment. When simulating bank stability, the shear strength parameters for the experiment soils under the two water contents are shown in Table 3.

#### 3.3.2. Validation of the Bank Failure Pattern

**Figure 10:**
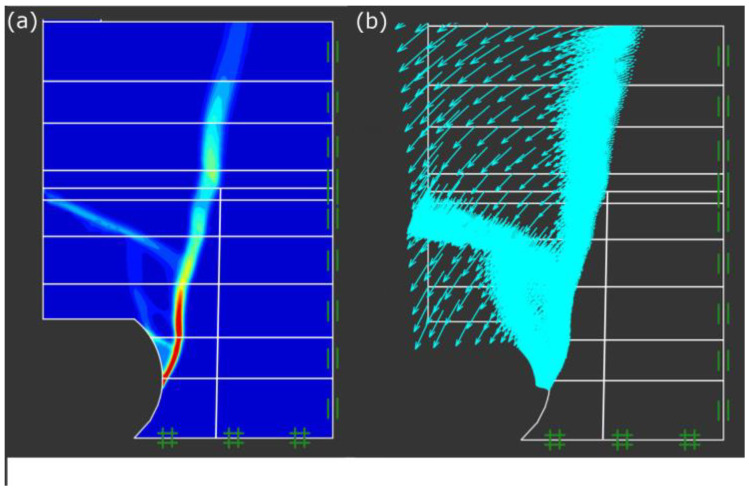
Validation result of the straight slope after scouring (under working condition 5): (**a**) the bank failure surface; and (**b**) displacement vector map.

The failure pattern of a straight bank after scouring (under test condition 5) was selected for validation using the OptumG2. The erosion height and width equal 0.39 and 0.29 m of the slope model in Figure 9, and the simulation results are stated in Figure 10.

The failure surface of the slope was almost vertical in Figure 10a. The simulated result was very similar to the failure mode of the scouring experiment; the upper suspended soil rotated and fell into the water under the action of gravity in Figure 10b.

#### 3.3.3. Validation of the Influence of Scour Width on the Bank Stability

**Figure 11:**
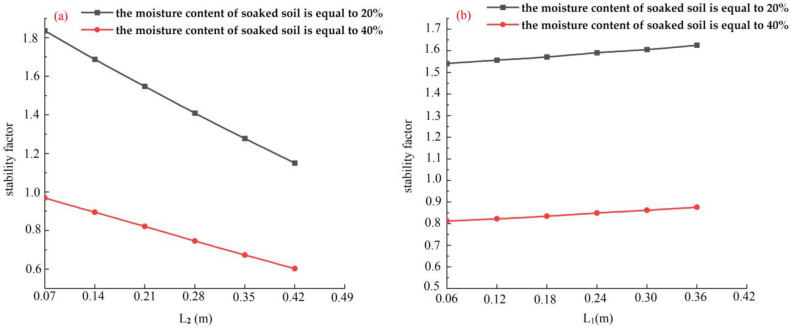
The effect of the scour degree on the bank stability: (**a**) the influence of the scour width on the stability coefficient; and (**b**) the effect of the scour height on the stability coefficient.

**Figure 12:**
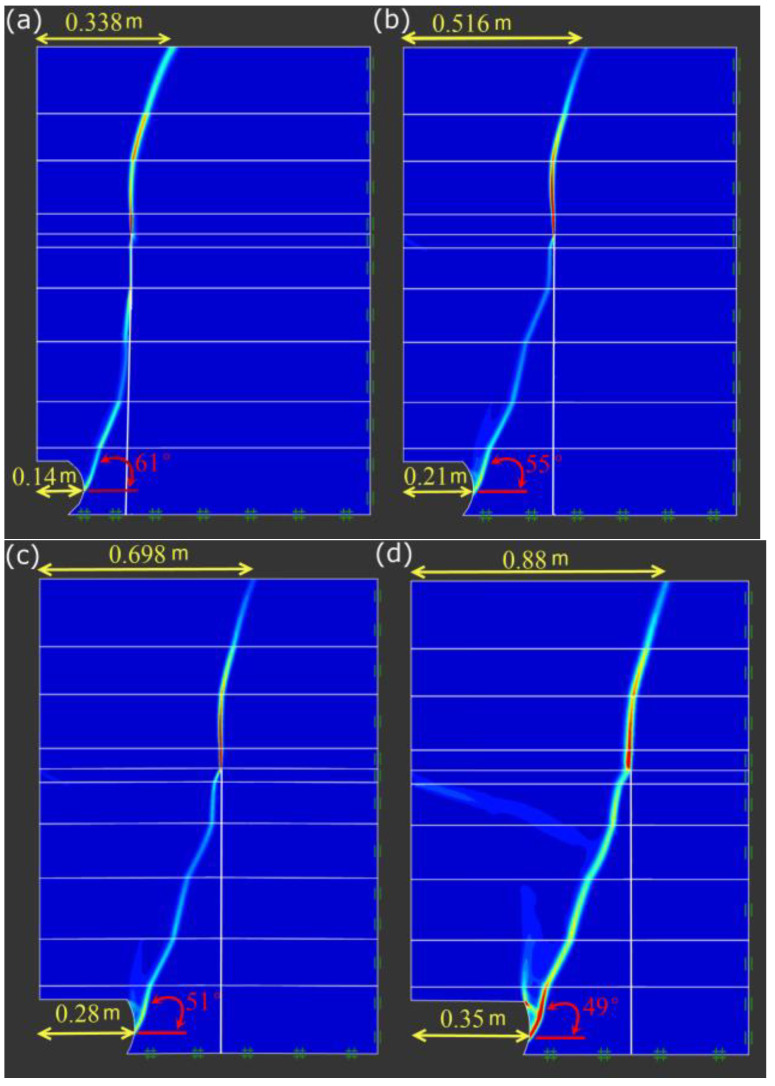
Variation of slope failure surface under four scour widths: (**a**) scour width is equal to 0.14 m, (**b**) scour width is equal to 0.21 m, (**c**) scour width is equal to 0.28 m, and (**d**) scour width is equal to 0.35 m (**a**).

**Figure 13:**
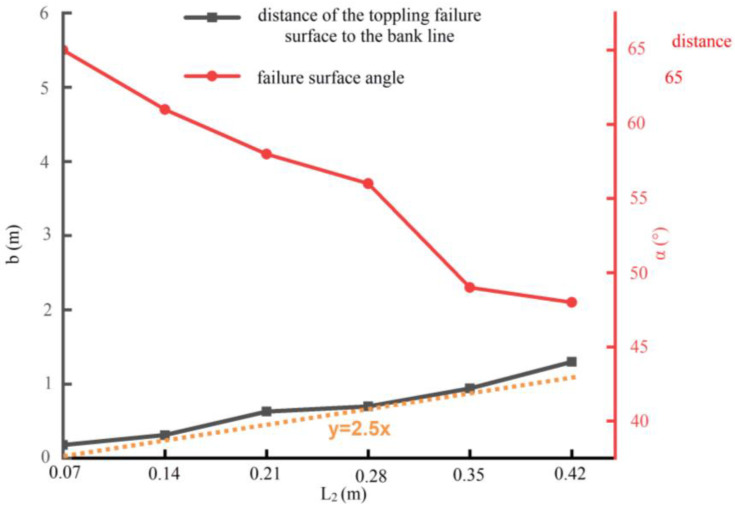
Variation in the angle of the failure surface and the length from the top failure surface to the bank edge with changes in scour width.

As the water flow continues to scour the slope, the scour width and height keep increasing, and the main reason for the bank collapse is severe local scour [69]. Therefore, it is critical to validate the influence of the scour width on slope stability. The bank model is shown in Figure 9, and the model parameters are shown in Table 4. The other variables remain unchanged when one variable changes. The simulation results are stated in Figure 11, Figure 12 and Figure 13.

The stability coefficient of the bank decreases significantly with an increase in the scour width in Figure 11a. This was consistent with the scour experiment in that banks first collapsed with large scour width when the scour height was almost the same. Contrary to the scour width, the stability coefficient of the bank increased slightly with an increase in the scour height (Figure 11b). The bank soil strength significantly declines when the water content increases from 20% to 40% (Table 4). The bank stability coefficient decreases rapidly under the dual effects of an obvious decrease in the soil strength and an increase in scour width ( Figure 11a).

When the scouring widths are 0.14, 0.21, 0.28 and 0.35m, respectively, the failure surface of the river bank is as shown in Figure 12. With an increase in the scouring width, the failure distance b of the shore top becomes larger, and the failure surface angle α gradually decreases. The processing results of b and α under six scour widths are shown in Figure 13. It is clear that α is negatively correlated with L_2_, and b is about 2.5 times that of L_2_.

## 4. Discussion

In the previous part of this paper, scour experiment and numerical simulations are combined to analyze the stability of interactive stratified slopes. In this part, the influence of water content and scour width on slope stability and bank failure law are discussed below.

In previous studies, water content was found to be an important factor affecting slope stability [27,28,29,30,31]. This article comes to the consistent conclusion from laboratory experiments that when the water content increased from 20% to 40%, the cohesion and internal friction angle of the clay decreased by about 60% and 67%, respectively, and the cohesion and internal friction angle of the silt soil decreased by about 61% and 43%, respectively (Table 4). Zhang studied the changes in water content and soil strength in the process of bank failure by using the numerical simulation method, and found that the water content increased rapidly in the initial stage; the change was relatively small in the later stage, and the soil strength of the bank decreased significantly [70]. The change in water content measured by Zhang is consistent with the results obtained by the earth pressure box in the scour experiment (Figure 8)which indicate that the variation trend in water content in multi-layer soil slope and single-layer soil slope is the same

According to the experimental observations, scouring is an important cause of bank failure. Numerical simulation results show that the bank stability coefficient decreased with the scour width increasing (Figure 11a). However, the stability coefficient of the bank increased slightly with an increase in the scour height. Zhang performed a systematic study on the failure of single-layer overhanging slopes and concluded that bank stability first decreases and then increases with an increase in erosion height [70]. This is because the stability of the sandy soil slope is poor, and the slope stability decreases rapidly after being soaked in water.

The slope was scoured to form an arc-shaped scouring surface under the action of water scour (Show in Figure 10), and the overhanging soil, broken through by the fissure, toppled when the bank slope was washed to a certain extent. Zhang summarized the failure pattern of single-layer bank slope and surmised that the length from the top failure surface to the bank edge is about twotimes of the scour width [70], which is smaller than the results of this paper (2.5 times). This difference may be caused by the clay layer in the stratified bank studied in this paper, which increases the integrity of the bank and makes the failure range of the top of the bank larger.

In this paper, failure law and the failure mechanism of floodplain slopes are studied through a flume experiment and numerical simulation. It is important to study the influence of scour width and water content on slope stability [27,28,70]. For now, only the general failure laws of the floodplain slope were obtained. The next step is to summarize the failure formulas which will also be calibrated with the bank failure data monitored in the field. Meanwhile, three-dimensional numerical simulation to study the erosion failure of the floodplain slope will be included in future research.

## 5. Conclusions

Scouring of water flow leads to rapid degradation of the floodplain which compresses the production space of the people and seriously impacts the environment. In this paper, the failure mechanism and laws of floodplain slopes are studied by combining flume experiments and numerical simulation. A total of five conditions were designed in the flume experiment, and the failure characteristics of concave banks, convex banks, and straight banks in all conditions were recorded and analyzed. Meanwhile, through the establishment of an equal-scale bank model, the bank failure results of the numerical simulation and erosion experiment were compared. The main conclusions are as follows.

The main reasons for the floodplains’ rapid collapse were that the slope toe was scoured, and the soil strength decreased rapidly; the bank stability coefficient decreased by about 65% when the scour width increased from 0.07 m to 0.42 m, and the water content increased from 20% to 40%.

The failure depth of the bank top is greatly affected by the scour width. It is found that the distance from the failure surface of the bank top to the edge, b, is about 2.5 times the scour width. Meanwhile, the failure surface angle diminishes with an increase in scour width.

Due to the rapid development of industry and agriculture on both sides of the river, the floodplain soil is seriously polluted, and floodplain slope collapse causes polluted soil to enter the river. The study of floodplain slope failure laws in this paper provides a new idea for the protection of the floodplain, and also lays a foundation for further study of the release law of pollutants in soil during floodplain slope failure.

## Figures and Tables

**Table 1:** The detailed arrangement of experimental groups.

Working Condition	Bank Height (cm)	Flow (L/s)	Bank Angle (°)	River Gradient	Scouring Time (h)
1	14	0.5	90	0.01	1
2	14	0.5	90	0.02	1
3	14	0.5	90	0.03	1
4	14	0.8	90	0.01	1
5	14	1.2	90	0.01	1

**Table 2:** Part test results of the brown soil.

Soil Label	Sampling Location	Depth (cm)	Moisture Content (%)	Dry Density (g·cm^−3^)	Wet Density (g·cm^−3^)	*Ip*	Shear Strength	
							*C* (kPa)	*φ* (°)
1	Wantantown	25	21.15	1.66	2.009	18.2	36.5	28.6
2	Wantantown	86	35.61	1.40	1.851	23.6	26.36	18.56
3	Heigangkou	30	16.7	1.57	1.836	21.1	49.1	32.3
4	Heigangkou	55	25.55	1.71	2.113	19.4	31.3	26.5
5	Liuyuankou	93	28.81	1.63	2.232	21.7	39.1	23.63
6	Liuyuankou	137	39.71	1.49	1.910	18.8	36.51	25.1

Symbols: *Ip* = Plasticity index.

**Table 3:** Model parameters for simulating the failure mode.

H (m)	H_1_ (m)	L_1_ (m)	L_2_ (m)	Moisture Content	Soil Type	*C* (kPa)	*φ*(°)	Dry Density (kN·m^−3^)	Wet Density (kN·m^−3^)
1.4	1	0.39	0.29	20%	clay silty sand	35 25	30 35	17 16	15 14
				40%	clay silty sand	15 10	10 20	18 17	15 14

**Table 4:** Model parameters for simulating the effect of the scour degree on the stability coefficient.

H (m)	H_1_ (m)	L_1_ (m)	L_2_ (m)	Moisture Content	Soil Type	*C* (kPa)	*φ*(°)	Dry Density (kN·m^−3^)	Wet Density (kN·m^−3^)
1.4	0.6 H	/	/	20%	clay silty sand	35 25	30 35	17 16	15 14
				40%	clay silty sand	15 10	10 20	17 17	15 14

## Data Availability

Data are available on request.

## Appendix A

**Table A1:** Soil sampling point locations.

Section Name	Latitude and Longitude	Sampling Depth (cm)
Wantantown Yangqiaovillage Heigangkou Nie zhuang Liuyunkou	113°56′41.08621″ 34°52′52.67728″	25, 63, 86, 113
	113°52′4.84872″ 34°51′54.74157″	13, 31, 58, 61, 123
	114°15′28.19616″ 34°53′42.83452″	28, 58, 65, 131
	114°16′0.72747″ 34°53′46.44162″	13, 39, 56, 113
	114°23′26.90973″ 34°54′34.64414″	16, 68, 85, 140

**Table A2:** Introduction of experiment equipment.

Experimental Equipment	Style	Equipment Role	Range of Parameter Variation
S-shaped flume	welded with the 1.5 mm thick iron sheet	Simulating the erosion process of the different banks	—
Flow meter	Electromagnetic flowmeter	Controlling water flow	0.5–1.2 L/s
Static strain tester	DH3818Y	Processing of earth pressure data	−4.0–5.0 pa
Earth pressure boxes	Vibrating string earth pressure box	Measuring the change in earth pressure during the experiment	—
Cameras	Sony Alpha7C	Capturing the slope failure process	—
Water baffle	Granite plate	Controlling the water level height by changing its position	—
Graduated scale	Steel rule	Measuring the digital height	5–10 cm
